# Semi-supervised classifier guided by discriminator

**DOI:** 10.1038/s41598-022-18947-6

**Published:** 2022-08-29

**Authors:** Sebastian Jamroziński, Urszula Markowska-Kaczmar

**Affiliations:** Department of Arificial Intelligence, Wroclaw University of Science and Technology, wyb. Wyspiańskiego 27, 50-30 Wrocław, Poland

**Keywords:** Medical research, Energy science and technology, Engineering, Mathematics and computing

## Abstract

Some machine learning applications do not allow for data augmentation or are applied to modalities where the augmentation is difficult to define. Our study aimed to develop a new method in semi-supervised learning (SSL) applicable to various modalities of data (images, sound, text), especially when augmentation is hard or impossible to define, i.e., medical images. Assuming that all samples, labeled and unlabeled, come from the same data distribution, we can say that labeled and unlabeled data sets used in the semi-supervised learning tasks are similar. Based on this observation, the data embeddings created by the classifier should also be similar for both sets. In our method, finding these embeddings is achieved based on two models—classifier and an auxiliary discriminator model, inspired by the Generative Adversarial Network (GAN) learning process. The classifier is trained to build embeddings for labeled and unlabeled datasets to cheat discriminator, which recognizes whether the embedding comes from a labeled or unlabeled dataset. The method was named the DGSSC from Discriminator Guided Semi-Supervised Classifier. The experimental research aimed evaluation of the proposed method on the classification task in combination with the teacher-student approach and comparison with other SSL methods. In most experiments, training the networks with the DGSSC method improves accuracy with the teacher-student approach. It does not deteriorate the accuracy of any experiment.

## Introduction

Classification is one of the common tasks that people try to solve using various automatic methods. These methods categorize samples into assumed categories based on the extracted features. Classification problems can refer to different data modalities, like images, video, text, or sound. The primary approach to pattern classification is to apply deep models because they offer automatic feature extraction; however, they need massive annotated datasets to train it. The quality of the model depends on how accurately data in the dataset is labeled. Manual annotations are precise but costly. Therefore other approaches are proposed^1^.

When a dataset is small, augmentation is frequently used. Data augmentation is a technique used to increase the amount of data by adding slightly modified copies of already existing data or newly created synthetic data from existing data. It acts as a regularizer and helps reduce overfitting when training a machine learning model. The use of the augmentation operations usually improves the quality of the model. It is relatively easy for natural images. In this case, augmentation includes geometric transformations, color space augmentations, kernel filters, mixing images, random erasing, feature space augmentation^2^. The disadvantage of augmentation is its need to adapt to the specificity of the input data. When we consider the natural language processing (NLP) domain, then more advanced and specific techniques must be applied^3^. Zhang et al.^4^ found that one of the valuable ways to do text augmentation is replacing words or phrases with their synonyms. For audio, we can apply noise injection, shifting time, changing pitch and speed, or nonlinear combination to construct new samples and linear method to construct labels^5^. For video, one considers augmentation on the spatial side, where the standard single-image augmentation strategies for image classification are used and on the temporal axis, when one randomly sub-samples a shorter video clip from the whole sequence^6^.

Another practical approach that is relevant when teaching models on a small amount of labeled data is transfer learning^7^. This approach uses a pre-trained model, i.e., a model trained on a larger, possibly similar, dataset. In this model, the last layers are replaced with new, randomly initialized layers. Then the parameters of new layers are trained on the available data while other parameters are frozen. Next, the whole network is tuned. Transfer learning gives better results when source tasks are related to target task^8^. In transfer learning, the body of the pre-trained network can be treated as a feature extractor or a function projecting the input data into a particular embedding space.

Data augmentation and transfer learning have a significant drawback when a task modality is restrictive, i.e., medical images. Data augmentation incorporates additional knowledge about the data that is not necessarily present in the data itself. When used on medical images, a common technique of horizontal image flipping might lead to training a predictive model that sees no anomaly in a patient with a heart located on the other side. Satellite image flipping might change the correlation between sunrise-sunset image features or features correlated with Coriolis Effect like hurricane spin. Similarly, the usage of transfer learning poses a risk of transferring any bias from the original domain to the final one. There are applications where the standard state-of-the-art techniques are sub-optimal or invalid to apply, and even a slight performance improvement is a significant step forward. We aim to develop a method for applications where such restrictions apply.

Recently, the somewhat old idea of pseudo labels included in semi-supervised learning (SSL)^9^ has been more and more popular in the case of deep learning^10,11^. SSL addresses training models with limited labeled patterns when a relatively large body of unlabeled patterns is available. The main goal of this task is to train a classifier model using both the unlabeled and labeled datasets. Ultimately, SSL allows for obtaining models with effects similar to the models trained in a fully supervised manner (Supervised Learning, SL), using a much smaller number of labeled samples based on the data representation extracted from unlabeled patterns.

Keeping in mind that

- complex data augmentation needs a lot of domain knowledge,
- it may be very difficult for some data modality^12^,
- possessing a huge annotated dataset is very expensive,

we wanted to avoid these problems. It was the main motivation to perform our research. It aims to develop a new SSL method applicable to image, text, sound, and possibly other data types. Its essence is not based on data augmentation, as it is in many current methods^13–15^. In^16^ authors demonstrated that the key to obtaining good SSL performances is the quality of the sample perturbations.

While building the method, we assumed that the representation of labeled and unlabeled data comes from the same data distribution, i.e. embeddings generated by hidden layers of the classifier network, should be similar, regardless of whether the classifier input came from a labeled or unlabeled dataset. Inspired by the Generative Adversarial Network (GAN) learning process^17^, we used an additional discriminator model and adversarial training to make labeled and unlabeled embeddings more similar. Such an approach allowed us to avoid the difficulty of defining the similarity measure of such embeddings. The proposed method was named Discriminator Guided Semi-Supervised Classifier, abbreviated to DGSSC.

Summing up, the main contribution of this research is a new method of semi-supervised learning (SSL) that can be characterized as follows:

- it profits from the assumption about the same data distribution of labeled and unlabeled datasets,
- it is not based on data augmentation,
- it can be applied to data of various modalities.

Most popular and best-performing SSL methods extensively use the augmentations and therefore incorporate external knowledge about the data in the training process. Our method stands out when compared to others as it applies to various challenging tasks. The method makes no assumptions about the data, which makes it completely independent of modality and the usage of augmentations.

The roots of our research lie in semisupervised learning, which is based on a small amount of labeled data with a large amount of unlabeled data used during training. Its primary goal is to avoid labeling a massive amount of data. The novelty of our method is the combination of adversarial training, first time introduced in GAN^17^ and SSL approach. Adversarial machine learning can be used in a variety of applications. This technique is most commonly used to execute an attack or cause a malfunction in a machine learning systems^18,19^. It is also applied in domain adaption, which refers to collecting the training and test sets from different sources or data shifts over time. In this case, there would be a discrepancy across domain distributions. At first glance, there is nothing in common with semisupervised learning and domain adaptation. However, considering unsupervised domain adaptation^20^ deeper, we can see that both problems are defined similarly. We have labeled data in the training set (source), and we want to predict labels for data in the testing dataset (domain). In this context, unsupervised adversarial domain adaptation methods will be interesting for comparison of similarity to our method. They minimize the distance between the target and source feature distributions (expressed by correlation distances or maximum mean discrepancy). The goal is to identify a feature space in which target and source domain samples are indistinguishable. We can mention here: CADA^21^, ACGAN^22^, DANN^23^, NoGRL^24^ methods. It is worth pointing out the differences of DGSSC in relation to them. The Consensus Adversarial Domain Adaptation method, abbreviated to CADA, is composed of four steps. In the second step, CADA uses an architecture that resembles the DGSSC architecture (two branches of the classifier for searching a domain-invariant feature space and discriminator). Still, both solutions differ in adversarial losses used to train the model. The Auxiliary GAN—ACGAN, unlike DCSSC, uses an adversarial image generation approach. DANN stems from Domain-Adversarial Neural Networks. The method does not use the idea of a discriminator. Instead, the authors introduce the gradient reversal layer (GRL) trick, ensuring that the feature distributions over the two domains are as indistinguishable as possible. In the NoGRL method, the gradient reversal layer is removed and substituted by the new confusion loss. The two last-mentioned methods differ in their nature compared to DGSSC.

The research described in this paper is contained in seven sections. In the next one, we give the background to the study by describing related works. The DGSSC method is shown in “Method”. Section “Experimental setup” describes experimental setup with the goal of experiments, datasets description, and experimental procedure. In section “Ablation study”, we analyze whether our assumptions align with the experiment. The central part of “Experiments” is focused on experimental results and discussion. The conclusions and plans for future research end the paper.

## Related work

In the introduction, we discussed the relationship of the DCSSG with adversarial domain adaption methods. This section will show state of the art in the SSL context. It is worth starting from the taxonomy of SSL methods proposed in^25^. It distinguishes two main trends: one focuses exclusively on labeling data not yet recorded (transduction approaches) and the other, which aims to develop a model that works for each new input data (inductive approaches). Then inductive methods are further split into unsupervised preprocessing, wrapper methods, and intrinsically semi-supervised.

Unsupervised preprocessing separates the classification task from representation (manifold) training. In the first step, the methods perform preprocessing in three ways by: unsupervised feature extraction, clustering, or supporting the initialization of weights of the target model. Only in the second step, they train the model in a supervised way using an annotated set. The primary example of feature extraction is searching data representation using Autoencoder^26^. In the NLP domain, we can mention the Masked Language Modelling method that learns to predict masked token representation based on other tokens in the sentence. They are very efficient in the fine-tuning models in the text classification^27,28^.

Intrinsically semi-supervised methods use unlabeled datasets directly in a loss function. It is opposed to unsupervised preprocessing methods where both datasets are treated separately. One category of intrinsically semi-supervised methods are generative models. They are characterized by teaching models that generate new data similar to the training data based on an unlabeled set. Modeling this process is possible thanks to General Adversarial Networks (GAN)^17^. They consist of two models: generator and discriminator. Training GAN is expressed by Eq. (1)

1 $$\begin{aligned} \min _G \max _D V(D, G), \end{aligned}$$

where *V* means the optimized function described by Eq. (2)

2 $$\begin{aligned} \begin{aligned} V(D, G)=&\mathbb {E}_{x\sim p_{data}(x)}[\log D(x)]\\&+ \mathbb {E}_{z\sim p_z(z)}[\log (1 - D(G(z)))], \end{aligned} \end{aligned}$$

*G* presents the function performed by generator while *D* refers to the function of discriminator. Various extensions are added to the discriminator in semi-supervised learning to consider labels into the function D^29–31^. In this new form, discriminator categorizes a given sample into K + 1 classes (K classes stem from the solved problem, and an additional class denotes the data coming from the generator). In such a form, the discriminator addresses the semi-supervised learning problem by using unlabeled data in the learning process.

Another worth mentioning example in this group is using Siamese networks. Training of Siamese nets needs the loss function based on the distance between the inputs embeddings. By default, in supervised learning (SL), this feature allows the SL method to approach the embeddings of samples from the same class while moving the embeddings of samples from other classes away by at least a certain distance (usually Euclidean or cosine) called margin. This technique of training models is called contrastive learning. The use of contrastive methods in SSL is based on a classifier that operates on the extracted data representation, as in the unsupervised pre-processing methods. Siamese network-based methods can be classified as manifold approximation methods^25^. Siamese-based methods often use substantial augmentation and a label propagation technique. An example is FixMatch^13^. In this case, to each unlabeled data classified above a certain threshold is assigned a pseudo-label. All labeled or pseudo-labeled data is heavily augmented and serves as data in contrastive learning.

By design, mixup methods are independent of augmentation. They imply fewer assumptions about the type of data they operate on, and in particular, do not require augmentation during their use in the SSL domain. The mixup technique is based on the linear interpolation of the training samples. An example of a method in the field of SSL is Interpolation Consistency Training (ICT)^32^.

Wrapping methods include training the teacher model T from labeled data, assigning pseudo labels to the data from the unlabeled set, and training the model student S on the obtained artificial labels. This process can be iterated assuming the previous model S as the new T model. It is teacher-student learning^11^. Examples that can be mentioned are the Noisy Student^33^ and self-distillation^34^ methods.

Readers interested in the taxonomy of deep semi-supervised learning methods based on loss function and model design are referred to^35^.

Graph based SSL methods rely on the geometry of the data represented by both labeled and unlabeled patterns. They create graph, where nodes correspond to training samples, edges represent similarities between them. It is possible to learn with very few labels how to propagate information through the graph by using the graph or manifold structure of data. These methods can be transductive as well inductive^25^. They consist of two steps: graph construction and label inference and can be further structured in each step. Details can be found in the survey paper^36^.

Our literature survey shows that SSL methods are mainly developed using one data modality, i.e., images (^13–15,36–38^), text (^39–43^) or sound (^12,44^). This unwritten standard runs the risk that developed approaches are effective only for this type of data. Many of the best-performing methods are based on the augmentation of data, which is modality-specific. This observation justifies the assumption made about building a generic method suitable for various modalities.

## Method

As we mentioned, the most effective SSL methods use augmentations or other transformations of the input data, introducing some knowledge about the data into the learning process. In our method, we want to make SSL techniques as much as possible independent of data augmentation to use it for various modalities (image, sound, text, etc.). Assuming that each training data from the labeled and unlabeled sets comes from the same data distribution, the embeddings (and predictions) generated by the model for labeled and unlabeled data types should also come from the identical distributions. This fundamental idea allows us to design our new method.

As the unlabeled and labeled samples are similar, i.e., come from the same distribution, all network activations on all layers should also be similar regardless if the sample is drawn from a labeled or unlabeled set. Also, we expect that if we used regular supervised learning with labeled data only, the network would likely overfit to samples seen during training. The overfitted model would make worse predictions for test samples than for the samples used for training, which means that the distribution of prediction vector values is different for training and test sets. Suppose the prediction vector values are of different distributions. In that case, we expect that we could notice distribution change for all network layers, on any depth, not only for the last prediction layer. We propose to pick a single layer of the classifier and use its activation distribution for the unlabeled points to detect when the overfitting occurs. More precisely, when the network is trained on a labeled set, we assume that if we monitor any layer activations for labeled and unlabeled sets, we could notice a growing distribution shift of the activations for labeled and unlabeled sets. We will call the activations of a chosen layer by embeddings. Further, we propose that if we encourage the embeddings for both sets to be more similar, we could somewhat prevent the overfitting. We notice that the training procedure of GAN networks is well suited for encouraging the embeddings of a labeled and unlabeled set to be similar.

**Figure 1:**
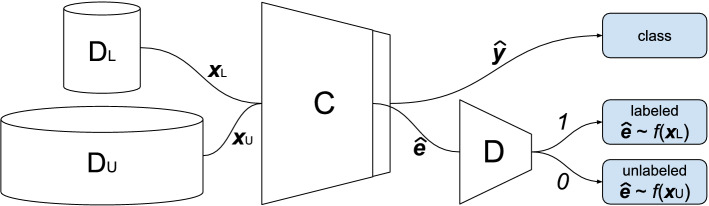
Data flow in the DGSSC method. $D_L$ denotes the set of labeled samples ${\textbf {x}}_L$, $D_U$ set of unlabeled samples ${\textbf {x}}_U$, *C* the classifier network and *D* the discriminator network. Classifier is trained in two modes. In supervised training it predicts output $\hat{{\textbf {y}}}$. It also takes a part in adversarial training to produce embeddings $\hat{{\textbf {e}}}$ of samples from both sets.

The DGSSC method combines a discriminator D known from the GAN network and a standard classifier C. Its idea is presented in Fig. 1. In other SSL methods based on GANs, the generator generates samples while the discriminator evaluates them. Then, the generator model is discarded after training. In our method, the discriminator is trained to distinguish between the embeddings $\hat{{\textbf {e}}}_L$ (for data stemming from the annotated set $D_{L}$) and the embeddings $\hat{{\textbf {e}}}_U$ of samples from the unlabeled set $D_U$.

The classifier’s role is to classify samples from $D_{L}$ dataset and to produce embeddings for samples from both datasets $D_{L}$ and $D_U$. The classifier is trained on the labeled set $D_L$ in a regular, supervised way. It is also trained to generate embeddings for unlabeled data so that the discriminator would not distinguish from which dataset the embeddings are (labeled or unlabeled). Classifier creates embeddings that can be taken from any layer. We considered embeddings from the last layer before using the Softmax function in the experiments.

The classifier is trained to find better and better representation (embeddings) for the unlabeled samples to cheat the discriminator that the given sample is from the $D_L$ dataset. The joint training of classifier and discriminator models encourages the improvement of classification performance.

Two loss functions are used during training the networks: $L_C$ for the classifier and $L_D$ for the discriminator. They are described in Eqs. (3) and (4), respectively.

3 $$\begin{aligned}&L_C = L_S + L_U \end{aligned}$$

4 $$\begin{aligned}&L_D = L_{D_L} + L_{D_U} \end{aligned}$$

The classifier loss $L_C$ in Eq. (3) is composed of the $L_S$ loss, which denotes a supervised learning part that updates *C* to classify labeled samples correctly, and the second component the $L_U$ loss, which refers to the unsupervised part. It leads *C* to treat unlabeled samples as labeled ones by changing *C* to produce embeddings in a way that would make *D* classify unlabeled samples as labeled ones. They are defined in Eqs. (5) and (6).

5 $$\begin{aligned}&L_S = H(\hat{{\textbf {y}}}_L, {{\textbf {y}}_L}) \end{aligned}$$

6 $$\begin{aligned}&L_U = H(D(\hat{{\textbf {e}}}_U), 1) \end{aligned}$$

In the above equations, *H* means cross-entropy, $\hat{{\textbf {y}}}_L$ classifier predictions for labeled set, ${\textbf {y}}_L$ true labels, ${\hat{{\textbf {e}}}_U}$ embedding of unlabeled sample, *D* represents the discriminator function. The discriminator recognises two classes—one corresponding to the labeled sample embedding when it outputs 1 and the second one for the unlabeled sample embedding when it outputs 0. In other words, 1 in Eq. (6) denotes target (label) used by discriminator for the labeled sample embedding.

The discriminator loss from Eq. (4) is composed of two components, as well. They are presented in Eqs. (7) and (8).

7 $$\begin{aligned}&L_{D_U} = H(D({\hat{{\textbf {e}}}_U}), 0) \end{aligned}$$

8 $$\begin{aligned}&L_{D_L} = H(D({\hat{{\textbf {e}}}_L}), 1) \end{aligned}$$

Variables here have the same meaning as in Eq. (6), i.e., *H* means cross-entropy, ${\hat{{\textbf {e}}}_U}$ embedding of an unlabeled sample, ${\hat{{\textbf {e}}}_L}$ embedding of a labeled sample. 0 and 1 denote targets (labels) used by the discriminator for the unlabeled and the labeled sample embedding, respectively. Moreover, *D* represents the discriminator function.

The $L_ {D_U}$ and $L_ {D_L}$ parts are used to train *D* to classify untagged and tagged samples, respectively. Even though in our case $L_U = -L_ {D_U}$, we decided to leave these terms under separate symbols because it better reflects the intuition behind the method. In this case, it is easier to imagine another choice of loss functions for *D* and *C* , for instance, leaving the cross entropy for the training *D* in $L_ {D_U} ^ * = H (D ({\hat{{\textbf {e}}} _ U}), 0)$ part and using mean square error for learning *C* in $L_U ^ * = MSE (D ({\hat{{\textbf {e}}} _ U}), 1)$.

Equivalently, we can express the loss function in the *minmax * form (Eqs. 9 and 10)

9 $$\begin{aligned}&\max _{C} [ - H(\hat{{\textbf {y}}}_L, {{\textbf {y}}_L}) + H(D(\hat{{\textbf {e}}}_U), 0)] = \max _{C} [ - L_S + L_{D_U}] \end{aligned}$$

10 $$\begin{aligned}&\min _{D} [ H(D({\hat{{\textbf {e}}}_L}), 1) + H(D({\hat{{\textbf {e}}}_U}), 0)] = \min _{D} [ L_{D_L} + L_{D_U}] \end{aligned}$$

where the adversarial setup arises from $H(D(\hat{{\textbf {e}}}_U), 0)$ term relating to Eqs. (6) and (7).

The procedure for updating classifier and discriminator weights is presented in Algorithm 1. As an input, the algorithm takes classifier and discriminator models and two batches of samples—labeled and unlabeled. Also optimizers $opt_C, opt_D$ for both models—classifier and discriminator are defined. As an output, the algorithm returns models of classifier and discriminator with updated weights.

This procedure is commonly referred to as a training step. It leads to updating models weights based on batches of samples drawn from labeled and unlabeled sets. When there are no more samples in one of the sets to pull the next batch, the set’s samples are reshuffled, and the next batch is drawn, independently of the second set’s state. The procedure is repeated until the monitored metric does not improve for the assumed number of consecutive measurements (in our experiments, it was equal to 8). This stop condition is commonly known as *early stopping*.

In Algorithm 1, lines 1 and 2 describe how the embeddings and predictions are calculated. Predictions stemming from unlabeled samples ($\hat{{\textbf {y}}}_U$) are not further used and are discarded. The loss calculation and weight update take place in lines 3-6 for the classifier, and in lines 7-10 for the discriminator.
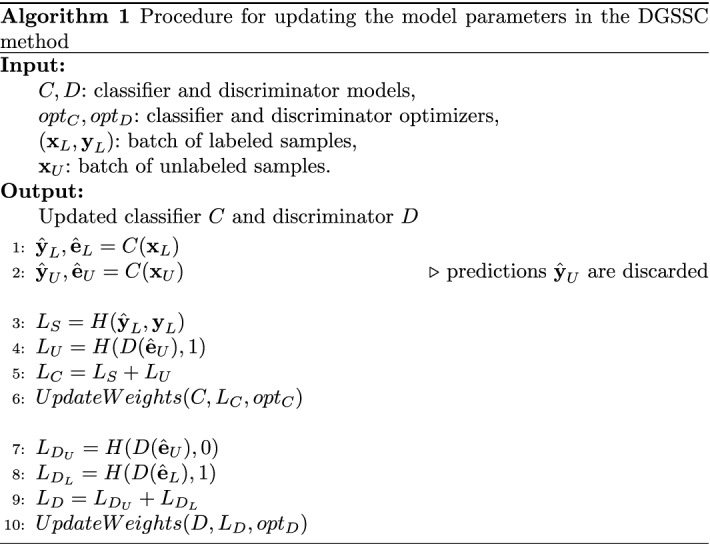


**Figure 2:**
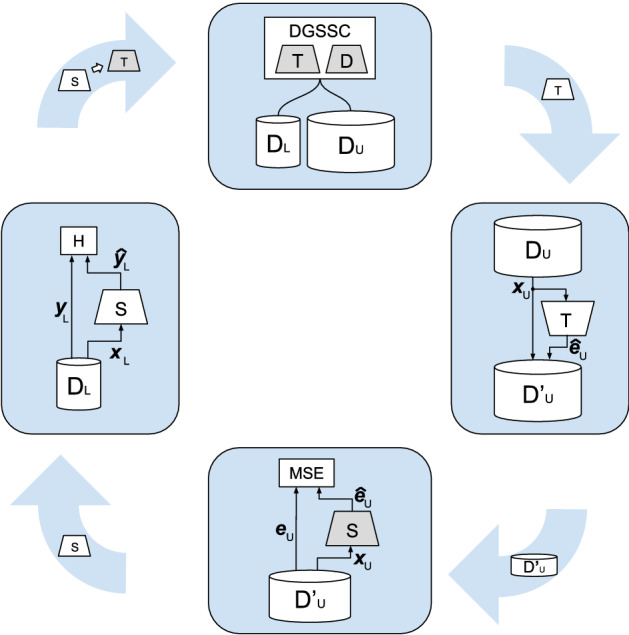
The diagram of model training in the teacher–student mode. The training comprises four main steps: starting from the one presented at the top—DGSSC procedure, followed by pseudo-labeling unlabeled set, knowledge transfer to a newly initialized model, and fine-tuning on labeled set. The objects on the blue arrows represent artifacts created by a given step used in the following step. The grey color denotes newly initialized models. *T* denotes the classifier–teacher, *D* discriminator, *S* the classifier–student, H cross-entropy loss, and MSE means squared error loss.

Similarly to^45^, we enriched the above learning process by the teacher- student approach. It is a procedure that generally leads to improvement in many SSL techniques. We decided to adopt it as it does not incorporate any additional knowledge about the data into the training procedure and allows the method to stay independent from the data modality. At the same time, it does not constrain the usage of modality-specific procedures (for instance, augmentations). Its idea is illustrated in Fig. 2. The proposed procedure consists of four main steps, which make up one cycle *O* of the teacher-student method. In the first cycle, the classifier–teacher *T* and discriminator *D* models are initialized. Then in the first step, they are trained using the DGSSC method showed in the top of the Fig. 2. In the next step of the cycle, the trained teacher *T* is used to achieve pseudo-labels—embeddings of unlabeled set $D_U$. It is represented by the rightmost element in Fig. 2. The new temporary dataset $D_U'$ contains: sample ${\textbf {x}}_U$, and the corresponding pseudo-label, i.e., embedding $\hat{{\textbf {e}}}_U$ assigned by the teacher network *T*. The dataset $D_U'$ can be expressed as follows:

11 $$\begin{aligned} D_U'=\lbrace<{\textbf {x}}_1,{\hat{{\textbf {e}}}}_1>,\ldots ,<{\textbf {x}}_P,{\hat{{\textbf {e}}}}_P>\rbrace \end{aligned}$$

where *P* is the number of samples in dataset $D_U$.

Next (bottom of Fig. 2), a new classifier-student *S* is initialized and trained to match its embeddings to teacher ones using the mean squared error loss function $L{_{MSE}}$ (in Fig. 2 assigned as MSE):

12 $$\begin{aligned} L{_{MSE}} = 1/p \sum _{i=1}^P\left\| {{\textbf {e}}_i}-\hat{{\textbf {e}}}_i\right\| ^2, \end{aligned}$$

where ${\textbf {e}}_i$ is the teacher embedding saved in the $D_U'$ set and $\hat{{\textbf {e}}}_i$ is the embedding of student classifier *S*. Lastly (the left element of Fig. 2) the trained student *S* is fine-tuned on the labeled set $D_L$ in a supervised way using cross-entropy loss $L_S$ defined in Eq. (5).

If it is the last cycle then the better classifier is returned, if not, student *S* becomes the new teacher model *T* and the cycle repeats without initializing a new teacher.

The detailed procedure of the teacher–student training scheme is presented in Algorithm 2. As an input, algorithm receives classifier $A_C$ and discriminator $A_D$ architectures, labeled $D_L$ and unlabeled $D_U$ datasets, the number of teacher–students cycles *O*, and the *DGSSC* method. As an output, it produces the trained classifier. In the first cycle, the classifier–teacher *T* and discriminator *D* are initialized (lines 3 and 5), and trained using the DGSSC method (line 6). The next step of the cycle is reflected in the line 7. Here, the trained teacher *T* is used to achieve pseudo-labels $\hat{{\textbf {e}}}_U$ of the unlabeled set $D_U$, and the pseudo-labels as the embeddings $\hat{{\textbf {e}}}_U$ with corresponding input ${\textbf {x}}_U$ are saved into temporary dataset $D_U'$ (line 7). The new classifier–student *S* is initialized (line 8), then trained to match its embeddings to teacher ones (line 9) using the mean squared error loss function (Eq. 12). The last step is described in line 10—the trained student *S* is fine-tuned on the labeled set $D_L$ in a supervised manner using cross-entropy loss. In the last cycle, the better classifier is chosen and returned. In the case it is not the last cycle, the student *S* becomes the new teacher *T* (line 11), and the cycle repeats without initializing a new teacher (line 3 is skipped).
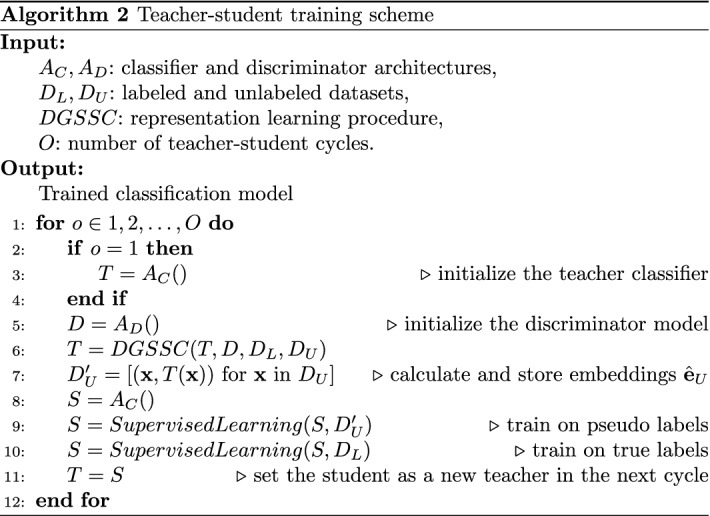


### Ethic

 We declare that this paper describes our original research, and it is not considered for publication in other journals.

## Experimental setup

The goal of the experimental research is to check whether the proposed method of training a classifier jointly with the discriminator leads to improvement in classification accuracy in SSL setup. We will evaluate the DGSSC method in combination with the teacher–student approach allowing for gradual propagation of labels over the unlabeled set. As a reference, a teacher–student approach with a supervised classifier in place of the DGSSC method will be used. A supervised baseline is a classifier trained only on the labeled set without using teacher–student nor the DGSSC method. Supervised classifier architecture is the same as the architecture of the classifier in the DGSSC method.

All experiments were performed using PyTorch library^46^ and PyTorch Lightning^47^, a framework organising PyTorch code. The hyperparameters not mentioned in the paper, for instance, weight initialization schemes, were left as defaults from the PyTorch library, including the sourcing of datasets. The experiments were conducted on a single GPU station (Intel i9 CPU 64 GB RAM 1$\times$ Nvidia RTX 3090 GPU).

### Datasets used

As we mentioned before, our goal was to verify whether the DGSSC method can be helpful for data in different modalities. Therefore in the experiments, we used six datasets of various modalities.

- *AG News*, a text domain dataset based on AG’s corpus of news articles in a form described by^4^. The dataset consists of 127, 600 short news from four categories: world, sport, business, and science/technology.
- *IMDB*^48^, a text domain dataset for binary sentiment classification. The dataset comprises 500, 00 movie reviews labeled as positive or negative.
- *Speech Commands*, a sound domain dataset introduced in^49^ consists of 95, 848 recordings of 25 words that are not uniformly distributed in the dataset.
- *FINDSOUNDS*, a sound domain dataset comprised of environmental sounds. The dataset was initially sourced from findsound.com search engine by the authors of^50^. It consists of 16, 930 sounds from 7 broad categories related to animals (e.g., cat, frog), people (e.g., coughing, laughing), nature (e.g., earthquake, ocean waves, flame), vehicle (e.g., car, helicopter, braking), noisemakers (e.g., alarm, bell, whistle), office (e.g., typing, printing) and musical instruments (e.g., bass, drum, synthesizer).
- *CIFAR-10*^51^, an image domain dataset. It is a typical dataset in the evaluation of SSL methods. It consists of 60, 000 $32 \times 32$ colour images from ten classes, with 6000 images per class.
- *SVHN*^52^, an image domain, digit classification dataset. It consists of 99, 289 $32 \times 32$ colour images of house numbers.

Detailed numbers of labeled, unlabeled, development and test dataset splits are presented in Table 1. All splits were stratified to preserve the relative frequency of class occurrences.

**Table 1:** Characteristics of the datasets used in the experiments. In the subsequent columns splits into labeled, unlabeled, development and test sets are given.

Dataset	No. of classes	The training set			Test set	Architecture
		Labeled	Unlab	Dev		
AG News	4	800	114,200	5000	7600	BERT
IMDB	2	400	19,600	5000	25,000	BERT
Speech Commands	35	4000	80,843	5000	11,005	M5
FINDSOUNDS	7	4000	6000	5000	1930	M5
CIFAR-10	10	4000	41,000	5000	10,000	CNN13
SVHN	10	4000	64,257	5000	26,032	CNN13

The last column describes the model architecture chosen for the corresponding dataset. All samples in dev and test sets are labeled, and predictions for these points are compared with their true labels to calculate the accuracy metric.

### Experimental procedure

During the training process to detect model improvements, the classification accuracy metric on the development dataset was monitored. The accuracy achieved using the test dataset is used to report the final evaluation of the proposed model. Formally, accuracy is defined as shows Eq. ().

13 $$\begin{aligned} accuracy = \dfrac{Number\, of\, correct\ predictions}{Total \,number\, of\, predictions} \end{aligned}$$

During the initial phase of method development, the following hyperparameters were proposed (they were not further optimised):

- shared in all stages:
  - *Learning rate decay:* learning rate is divided by 10 after 5 consecutive validation measurements without improving the monitored metric,
  - *Early stopping:* if the monitored metric does not improve for 8 consecutive measurements, the training process is stopped.
  - *Batch size:* it is set to 100 based on the value proposed in^32^,
  - *Validation measurement:* it is performed on unlabeled dataset after every epoch in order to avoid ambiguity of epoch length of labeled and unlabeled sets,
- optimizers:
  - *Adam* with $lr=0.001$, $\beta _1=0.9$, $\beta _2=0.999$, default from the PyTorch library, for the transfer of knowledge while training models on pseudo-labels in teacher-student mode,
  - *SGD* with $lr=0.1$, $momentum=0.9$, $weight\_decay=10^{-4}$, as proposed in^32^, for the DGSSC classifier and baseline classifiers,
  - *SGD* with $lr=0.1$, $momentum=0$, $weight\_decay=0$, default from the PyTorch library, for the DGSSC discriminator.

In the method development phase, while working on the CIFAR-10 dataset, the three presented combinations of optimizers were tested for each of the training steps of the teacher-student model and baseline classifier, and the best ones were adopted for further experimentation.

Experiments on the text modality were performed using BERT architecture^28^. Its details are described in “Model used”. It is a relatively large and slow to train model, which forced us to reduce some of the hyperparameters as follows:

- *Batch size:* it was set to 10 due to limitations of our GPU memory,
- *Learning rates:* they have been divided by the factor of 100 for each optimizer to account for the smaller *batch size*, i.e.:
  - *Adam* with $lr=1\text {e}-5$ for the teacher–student mode,
  - *SGD* with $lr=0.001$ for the DGSSC classifier and baseline classifiers,
  - *SGD* with $lr=0.001$ for the DGSSC discriminator.
- *Validation measurement:* it is performed after processing every 4000 sample from labeled and unlabeled sets, as awaiting for the processing of complete unlabeled set was infeasible because of computing resources.

In the case of text modality, remaining hyperparameters (for example *early stopping* or other optimizers’ hyperparameters) were left unchanged for this modality. In the development phase, we experimented with the division of all *learning rates* by the factor of 100 or 1000.

Experiments for all datasets in this section were repeated for different splits of the training dataset into labeled, unlabeled, and development datasets. There were three splits for the text modality and five for other modalities. Experiments on all datasets were made without using augmentation techniques.

In the case of the CIFAR-10 dataset, for comparison purposes with methods that use augmentation, we also did the augmentation in the form of zero padding by 2 pixels followed by random cropping back to the $32\times 32$ resolution and random horizontal flip with probability 0.5. The augmentations are adopted from^32^ and are used solely for better comparison with other methods.

We used 10 cycles in the teacher-student mode of training except for text modality where we train models for only 3 cycles as training large models based on BERT architecture was far slower compared to other experiments.

### Model used

In DGSSC, two models play an essential role—discriminator *D* and classifier *C*. The discriminator model consists of five hidden layers of ten neurons with ReLU nonlinearity and one layer projecting to a scalar with Sigmoid nonlinearity. The architecture of ten hidden neuron layers was assumed to be both expressive enough and resulting in low model size as to make experiments fast. The discriminator input values were sorted as it was deemed to simplify the discrimination task as argued in the Ablation Study section.

The proposed method is independent of deep neural network architecture used as the classifier model. The choice of the classifier model depends on the problem domain/modality (image, text, sound).

As a text classifier we used the pretrained BERT model^28^ as proposed in^42^. It is a large model compared to models used in our experiments for other modalities. Detailed description of model layers is presented in Table 2. Training BERT model forced us to make changes in hyperparameters, primarily the *batch size* and corresponding *learning rate* to allow training the model on our station. We also reduced other hyperparameters influencing training time, such as the number of teacher–student cycles and frequency of measurements used for *early stopping*.

**Table 2:** BERT architecture.

Layer description	Output size	#Neurons
Input—token Ids (T)	T	
Encoder—words 30,522, pos. 512, tokens 2, dim. 768	Tx768	23,837,184
$BERT_{BASE}$ – L: 12, H: 768, A: 12	Tx768	85,054,464
AvgPool	768	
Linear—128	128	98,432
Linear—num. classes	C	128C $+$ C
Softmax	C	

T denotes the maximum length of a sample for a given task, i.e. $T=250$ for IMDB and $T=70$ for AG News datasets, C indicates the number of classes for a given task, i.e. $C=2$ for IMDB and $C=4$ for AG News datasets.

For the sound classification, the M5 network^53^ was used. It is a 1D-CNN model used commonly for the Speech Commands dataset in the PyTorch ecosystem. Detailed description of the model architecture is presented in Table 3.

**Table 3:** M5 architecture.

Layer description	Output size (SpeechCommands or FindSounds)	#Neurons
Input (16kHz)—one channel, length L	16,000 or 40,000	
1Dconv—32, kernel 80, stride 16 + batch norm	32 $\times$ 996 or 32 $\times$ 2496	2656
MaxPool1D—kernel 4	32 $\times$ 249 or 32$\times$ 624	
1Dconv—32, kernel 3, stride 1 + batch norm	32 $\times$ 247 or 32 $\times$ 622	3168
MaxPool1D– kernel 4	32 $\times$ 61 or 32 $\times$ 155	
1Dconv—64, kernel 3, stride 1 + batch norm	64 $\times$ 59 or 64 $\times$ 153	6336
MaxPool1D—kernel 4	64 $\times$ 14 or 64 $\times$ 38	
1Dconv—64, kernel 3, stride 1 + batch norm	64 $\times$ 12 or 64 $\times$ 36	12,480
MaxPool1D—kernel 4	64 $\times$ 3 or 64 $\times$ 9	
AvgPool1D	64 $\times$ 1 or 64 $\times$ 1	
Linear—num. classes	35 or 7	2275 or 455
Softmax	35 or 7	

For the image classification, the CNN13 network was used as proposed in^32^. Detailed description of the model is presented in Table 4.

**Table 4:** CNN13 architecture. Every layer weights are stored in normalized form as proposed in^54^, i.e. weights are decomposed to magnitude and direction components.

Name	Layer description	Output size	#Neurons
	Input—32 $\times$ 32 image	3 $\times$ 32 $\times$ 32	
$layer_0$	3 $\times$ 3conv—128, padding 1 + batch norm	128$\times$ 32$\times$ 32	3968
$layer_1$	3 $\times$ 3conv—128, padding 1 + batch norm	128 $\times$ 32 $\times$ 32	147,968
$layer_2$	3 $\times$ 3conv—128, padding 1 + batch norm	128 $\times$ 32 $\times$ 32	147,968
	2$\times$2MaxPool, stride 2, padding 0	128 $\times$ 16 $\times$ 16	
$layer_3$	3 $\times$ 3conv—256, padding 1 + batch norm	256 $\times$ 16 $\times$ 16	295,936
$layer_4$	3 $\times$ 3conv—256, padding 1 + batch norm	256 $\times$ 16 $\times$ 16	590,848
$layer_5$	3 $\times$ 3conv—256, padding 1 + batch norm	256 $\times$ 16 $\times$ 16	590,848
	2 $\times$ 2MaxPool, stride 2, padding 0	256 $\times$ 8 $\times$ 8	
$layer_6$	3$\times$ 3conv—512, padding 0 + batch norm	512 $\times$ 6 $\times$ 6	1,181,696
$layer_{7}$	3 $\times$ 3conv—256, padding 0 + batch norm	256 $\times$ 6 $\times$ 6	132,096
$layer_{8}$	3 $\times$ 3conv—128, padding 0 + batch norm	128 $\times$ 6 $\times$ 6	33,280
$layer_{9}$	6 $\times$ 6AvgPool, stride 2, padding 0	128	
$layer_{10}$	Linear—10	10	1300
$layer_{11}$	Softmax	10	

Column *name* denotes a layer name used in this paper’s, where activations of different layers are analyzed.

## Ablation study

Before presenting the experiments, to show intuitions standing behind the idea of our method in this section, we describe a simple example with the CNN13 network, which we call the baseline classifier. This network was trained in a supervised way using 4000 labeled samples from the CIFAR10 dataset (without augmentations). We analyzed the network activations on subsequent layers for 1000 from 4000 labeled samples used for training and another 1000 unlabeled ones (unseen by the classifier during training).

**Figure 3:**
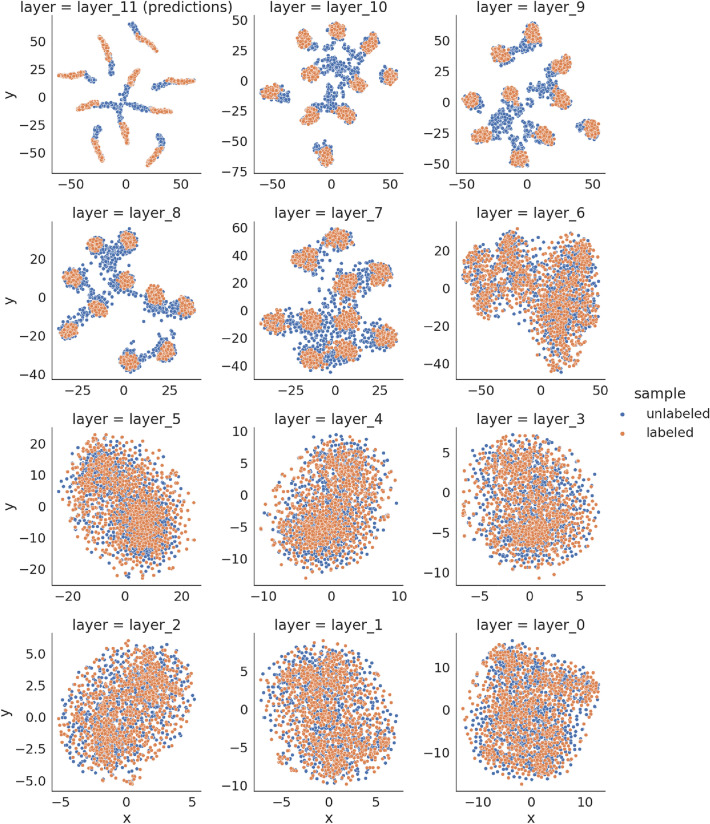
T-SNE projection of activations of hidden layers for labeled and unlabeled samples for baseline classifier trained for CIFAR-10 dataset. From top-left shown are the predictions ($layer_{11}$), logits ($layer_{10}$), and other intermediate layers until the projection of activations of the first convolutional layer (bottom-right, $layer_0$). Labeled and unlabeled samples come from the same distribution therefore their activations should be indistinguishable, which is not satisfied in this case.

In Fig. 3 we present the network activations from subsequent layers projected to 2 dimensional space using T-SNE method (the parameters of T-SNE method were left as defaults from scikit-learn python package^55^: $perplexity=30.0, learning\_rate=200$, and *random* initialization), marked as labeled (orange) or unlabeled (blue).

We can spot significant differences in how the network activations look like for labeled and unlabeled samples for layers from $layer_7$ to $layer_{11}$. When we assume that labeled and unlabeled samples come from the same distribution, their activations should be indistinguishable, which is not satisfied in this case.

The smaller the discriminator input, the faster the training is. Therefore we decided to use activations ${\textbf {e}} \in R^{10}$ of $layer_{10}$ as discriminator input. We will refer to them as sample embeddings. Inspired by the adversarial training from GAN^17^, we decided to use an auxiliary discriminator to encourage the embeddings for both labeled and unlabeled samples to be indistinguishable.

The model overfits to labeled samples used for training, resulting in the predictions for samples used for training having in general one value very close to 1 and the rest close to 0. The trained model makes less confident predictions for unlabeled samples, resulting in the highest value of the prediction vector being lesser in general than for the overfitted (labeled) samples, and probability mass will spread on other dimensions.

Let us notice that to recognize the sample as labeled one, the relationship between components of the prediction vector is essential. Its one value assigning the class should be almost equal to one, while others should be low. To distinguish the labeled samples from unlabeled, assigning to which class the sample belongs is not essential. Instead, it is crucial to identify this relationship between components of the prediction vector. In other words, rather than recognizing if an unlabeled sample belongs to one of 10 clusters formed by labeled sample predictions near points $(1,0\dots 0), (0,1\dots 0) \dots (0,0\dots 1)$ in the prediction space, it is better to identify whether an unlabeled sample falls into a single cluster.

**Figure 4:**
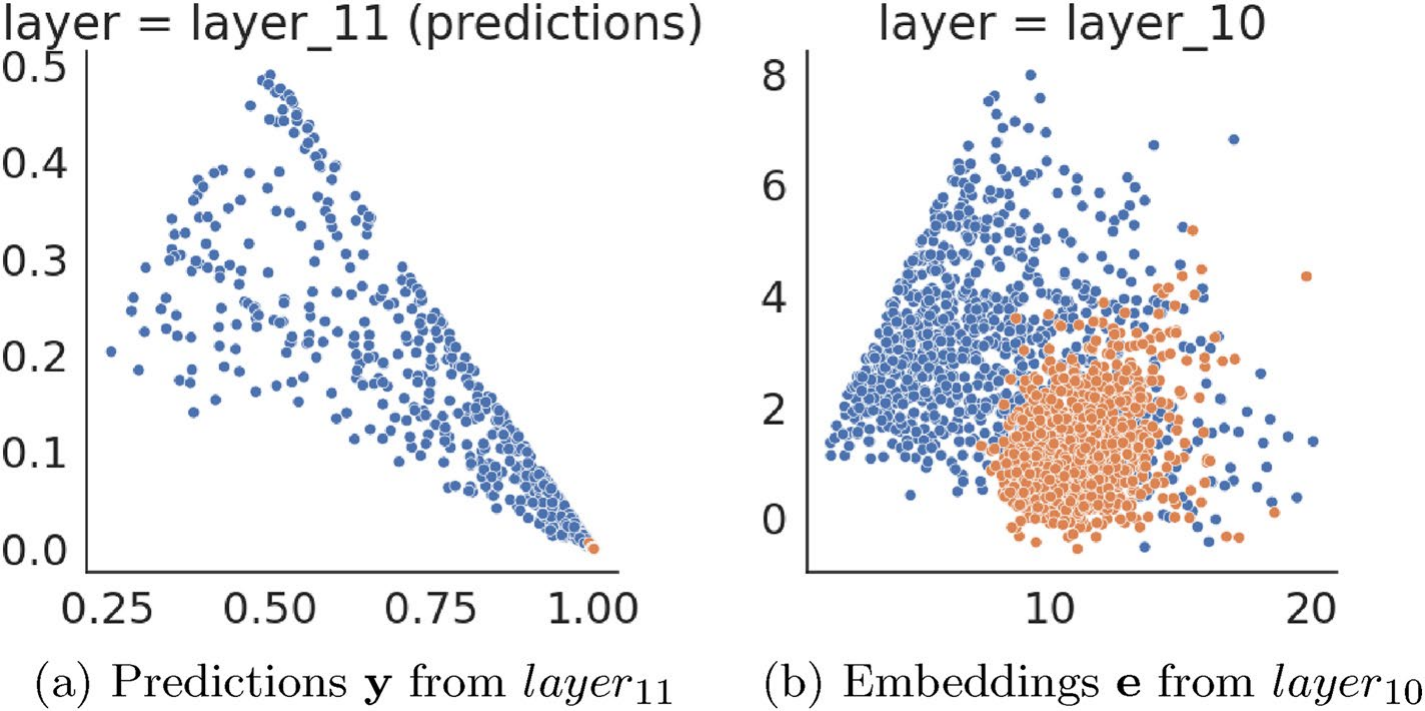
Two greatest components of activations for labeled (orange) and unlabeled (blue) samples for baseline classifier trained for CIFAR-10 dataset. Labeled samples (orange) are drawn on top of unlabeled ones (blue).

Figure 4a presents the scatter-plot of two most significant component values for labeled and unlabeled sample predictions $\hat{{\textbf {y}}}$, which confirms our reasoning—many unlabeled sample predictions are easily separable from labeled overfitted ones. The last layer of all models considered in this paper is the Softmax operation, which preserves the ranking of its input values. We therefore suspect that $layer_{10}$ embeddings ${\textbf {e}}$ may behave similarly to $layer_{11}$ predictions $\hat{{\textbf {y}}}$ under the sorting operation. As presented in Fig. 4b, we can notice that this is true as the labeled sample embeddings form a single cluster by observing the two most significant values of the embedding. This approach enables the discriminator model to operate on sorted embeddings and radically facilitates the task for the discriminator.

**Figure 5:**
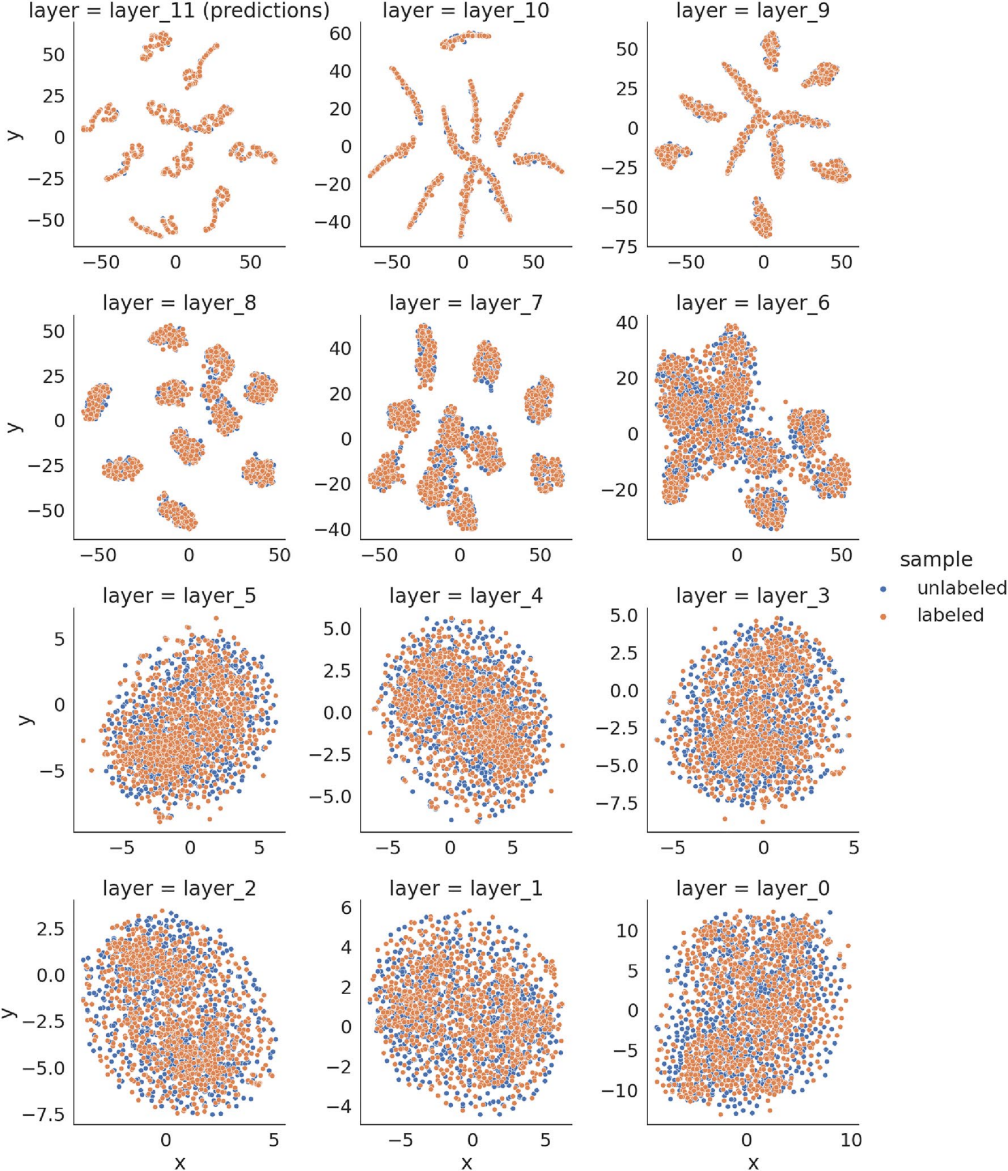
T-SNE projection of activations of hidden layers for labeled and unlabeled samples for classifier trained using the DGSSC method for CIFAR-10 dataset. From top-left shown are the predictions ($layer_{11}$), logits ($layer_{10}$), and other intermediate layers until the projection of activations of the first convolutional layer (bottom-right, $layer_0$).

Training using our method led the classifiers activations to be more similar for labeled and unlabeled samples used in the training procedure. Fig. 5 presents T-SNE projection of classifier activations trained on the CIFAR-10 dataset (without the use of augmentations). Model activations for labeled and unlabeled samples from the last layers are noticeably more similar than in the case of the baseline classifier. More precisely, T-SNE projection of activations from $layer_7$ forwards for the labeled and unlabeled samples are similar for the model trained with our method. Using the baseline classifier, the projection of unlabeled samples’ activations is more spread than labeled samples.

To summarize, we can present the idea more formally, given the above intuition standing behind our approach. All problems considered in this paper refer to the multiclass classification; therefore, the labeled sample ${\textbf {x}}_{\textbf {i}} \in {\textbf {D}}_{\textbf {L}}$ has the output prediction of the Softmax layer - ($layer_{11}$) in the form of vector ${{\hat{{\textbf {y}}}}_{\textbf {i}}}=[{\hat{{\textbf {y}}}}_{i1},{\hat{{\textbf {y}}}}_{\textbf {i2}},...,{\hat{{\textbf {y}}}}_{\textbf {ic}}]$, such that to following formulae is true:

14 $$\begin{aligned} \exists !_{k: k\in \{1,...,c\}}{\hat{y}}_{ik}\approx 1 \wedge \forall _{j:j\ne k \wedge j \in \{1,...,c\}} {\hat{y}}_{ij}\approx 0; \end{aligned}$$

where *c* is the number of classes. This relationship between the prediction vector components is valid for all labeled samples, independently of which *k* (which class) it is.

Having in mind that the set $D_L$ is relatively small, with high probability the network is overfitted. Therefore considering embedding vector ${\varvec{{\hat{e}}_{\textbf {i}}}}=[{\varvec{{\hat{e}}_{\textbf {i1}}}},{\varvec{{\hat{e}}_{\textbf {i2}}}},\ldots ,{\varvec{{\hat{e}}_{\textbf {ic}}}}]$, for the same sample ${{\textbf {x}}_{\textbf {i}}}$ from $layer_{10}$, for embedding vector components the relationship shown in Eq. (14) can be expressed as follows:

15 $$\begin{aligned} \exists !_{k: k\in \{1,\ldots ,c\}}{\hat{e}}_{ik}>>{\hat{e}}_{ij;\forall _{j: j\ne k;j\in \{1,\ldots ,c\}}}. \end{aligned}$$

This relationship, independently on *k*, characterizes the set of the labeled dataset and allows the discriminator to distinguish between labeled and unlabeled datasets.

To make the task for discriminator easier, we reorder the components of the embedding vector by sorting its components. For distinguishing labeled samples, it is not essential on which position is the highest value of the component.

Next, during adversarial training, the embeddings of unlabeled samples become more and more similar to the labeled ones, i.e., the relationship from Eq. (15) between components of the embedding vector arrives for unlabeled samples as well.

## Experiments

**Figure 6:**
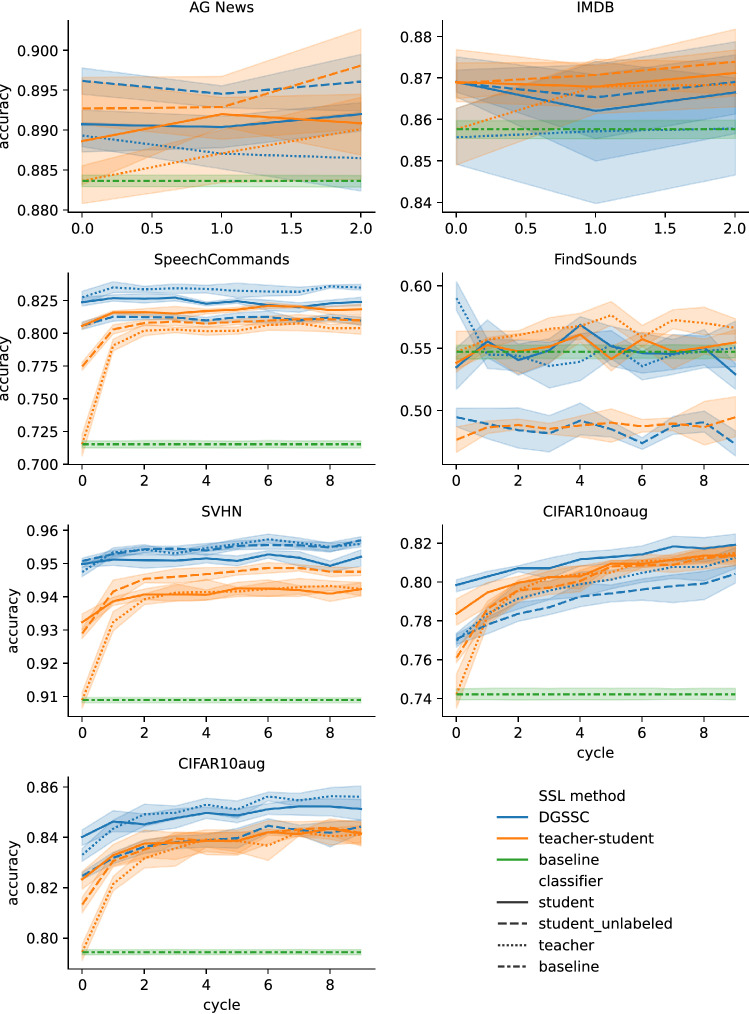
Experimental results of the proposed method and reference baselines. The lines represent the average from runs of each experiment and the lighter regions represent the standard deviation of the aggregated runs.

In order to validate the influence of the DGSSC method on classification accuracy in a semi-supervised setup, a supervised classifier in place of the DGSSC method was used. The results of the training procedure are presented in Fig. 6 and Table 5.

**Table 5:** Classification accuracy of the proposed method under different configurations, reference baselines and reference from other works.

method	AG News	IMDB	Speech Commands	FindSounds	SVHN	CIFAR-10	CIFAR-10 + augm
DGSSC (no teacher–student)	88.93±0.15	85.56±0.67	82.75±0.61	59.02±1.48	94.75±0.17	77.00±0.47	83.32±0.35
DGSSC (cycle 0)	89.61±0.16	86.90±0.53	82.75±0.61	59.02±1.48	95.08±0.10	79.86±0.42	84.02±0.41
DGSSC (last cycle)	89.61±0.36	86.90±0.89	83.49±0.27	55.04±2.31	95.71±0.14	81.93±0.67	85.62±0.58
Supervised baseline	88.36±0.25	85.76±0.75	71.53±1.10	54.72±2.34	90.90±0.31	74.22±1.27	79.45±0.39
Teacher–student only (without DGSSC)	89.81±0.69	87.39±1.11	81.84±0.56	56.63±0.84	94.76±0.16	81.50±0.54	84.18±0.59
MixText^42^	89.2	89.4					
MixMatch^14^					97.11±0.06		95.05±0.08
ICT^32^							92.71±0.02
FixMatch^13^							95.74±0.05

Each result of our experiments is provided with the mean and standard deviation calculated from 3 experiment runs for AG News and IMDB datasets, and 5 runs for other experiments.

The subsequent rows of Table 5 show results of the DGSSC method without the teacher–student approach, the teacher–student approach with DGSSC representation learning at the first and last generation, the teacher–student approach with the supervised classifier replacing the DGSSC method, the supervised classifier baseline trained only on the labeled dataset and results of other methods. The results for the pure DGSSC method are equivalent to the performance of the first generation teacher when combining the method with the teacher–student approach. Subsequent columns in this table refer to various datasets.

The DGSSC method outperformed the reference baselines on datasets: *Speech Commands*, *SVHN* and *CIFAR-10* with augmentations. No significant difference over the teacher–student-only approach was found on other datasets. In the case of non-image modalities, the usage of the teacher–student method did not improve the accuracy within consecutive cycles.

The analysis of the *Speech Commands* results shows that training with our method quickly reached its highest score (in the second cycle). In contrast, training without our method took approximately 4 cycles in the teacher–student mode to get the final score. We suppose that the characteristic of datasets plays an essential role in the training speed in terms of teacher–student cycles. The final score can be reached even in the first cycle, depending on the task.

Figure 6 visualizes the results for each cycle during training (horizontal axis). The vertical axis presents accuracy measured on the test dataset. The blue lines show experimental results of training DGSSC model combined with the teacher-student approach. The orange lines show the teacher-student approach with the supervised classifier in place of the DGSSC method. The green line represents the supervised baseline classifier. Dotted, dashed, and continuous lines represent the teacher, student trained on pseudo-labeled set, and the final student classifier results, respectively.

In the case of experiments on the CIFAR-10 without the use of augmentations, FindSounds, AG News, and IMDB datasets, the best results of our method overlap with the teacher-student baseline (teacher-student with supervised classifier in place of the DGSSC method), which is visible as overlapping error bands in Fig. 6. Out of those datasets, we see improvements only on CIFAR-10 without the use of augmentations and FindSounds datasets, where our method outperforms teacher-student baseline only in the first cycle of teacher-student training. The last four rows of Table serve as a general view of what is possible with the same combination of a dataset and labeled-unlabeled split. As noted in^56^, a direct comparison between the performance of different implementations shall not be made. With this in mind, we compare ourselves to MixText^42^ on text modality. We notice that our baselines and method (with nearly the same accuracy) achieved state-of-the-art results on AG News for 200 labeled samples per class. It is worth noting that experiments in the MixText paper were performed using only 5000 unlabeled samples during training which is significantly less than in our experiments. We decided to use all available unlabeled samples as it better refers to the SSL task (the SSL assumptions state that unlabeled samples are intrinsically available and the main cost of sourcing the data is the labeling process) and to be consistent with experiments on all other modalities. For the sound data classification, we found no adequate papers to compare. As a best effort, we can refer to^50^. Here, authors report *Unweighted Average Accuracy* (UAR) of $64.8\pm 1.6\%$ on *FindSounds* dataset with the use of 4000 labeled examples. Our experiments achieved (UAR) $27.17\pm 0.41\%$ for this dataset. For image modality, we compare ourselves to MixMatch^14^, ICT^32^, and FixMatch^13^ all of which outperform our results. Yet, given enough hyperparameter tuning and incorporating the same priors (i.e., in the form of adequate augmentation strategy), we suppose that our method would perform on par with those methods.

The results show that our method is promising in case of a challenging task, i.e., if it is hard to define augmentation or otherwise incorporate knowledge about the data. The proposed hyperparameters are reasonably robust to domain change, and it is unlikely that the usage of our method will degrade the final performance of a custom task. Further task specific fine-tuning would likely improve the performance.

## Conclusion and future work

Improvements of the proposed method are smaller than the best-performing state-of-the-art methods. However, those methods usually require extensive use of augmentations or, in other ways, incorporate knowledge about the data that is not necessarily present in the data itself. Our method is likely to improve results and is unlikely to introduce any bias into the model that would stem from assumptions required by other methods. We think the DGSSC method is beneficial for restrictive tasks requiring careful control over data assumptions.

In many experiments, training the networks with the DGSSC method improves accuracy when combined with the teacher–student approach of knowledge transfer. It does not deteriorate the accuracy in any of the experiments. The experiments involved two datasets per modality, single model architecture per modality, only one labeled-unlabeled split ratio per dataset, and datasets of different magnitudes. Therefore, it is impossible to conclude any significant insights on why the method performs on par with the teacher–student only method in some cases. As research requires intensive computing, we will leave this issue for clarification in the near future.

For the same reason, we conduct the experiments on the DGSSC method without tuning hyperparameters to the specific architecture and dataset combination. Further experiments with more computing resources could lead to more insight into the practicality of the method. The results suggest that even a single set of hyperparameters is enough to improve the accuracy for a wide range of applications.

The current version of our method leaves place for further improvements. Our nearest plans are listed below:

- evaluation of the proposed method with the discriminator working on embeddings taken from different depths (layers) of the classifier networks,
- swapping the discriminator with nonparametric loss functions such as Kullback–Leibler divergence,
- usage of different cost functions in the knowledge transfer in the teacher–student approach (in this paper, only euclidean distance has been applied)

Finally, we would like to leave the reader with a thought that enforcing similarity of intermediate features between labeled and unlabeled samples in SSL is worth attention. We see the idea of leveraging invariance of model activation distribution by using adversarial training as a modality agnostic analog of the idea of leveraging data-specific invariance by using augmentations.

## Data Availability

FindSounds dataset is available from the URL provided in^50^: http://zenodo.org/record/61295. All other datasets were sourced from the references provided by Pytorch^47^ library. SpeechCommands dataset: https://pytorch.org/audio/stable/datasets.html, text datasets: https://pytorch.org/text/stable/datasets.html, image datasets: https://pytorch.org/vision/stable/datasets.html.
